# Relationships between adverse childhood experiences and adult mental well-being: results from an English national household survey

**DOI:** 10.1186/s12889-016-2906-3

**Published:** 2016-03-03

**Authors:** Karen Hughes, Helen Lowey, Zara Quigg, Mark A. Bellis

**Affiliations:** Centre for Public health, Liverpool John Moores University, 15-21 Webster Street, Liverpool, L3 2ET UK; Blackburn with Darwen Borough Council, Public Health Department, 10 Duke Street, Blackburn, BB2 1DH UK; Bangor University, Normal Site, Bangor, LL57 2PZ UK; Director of Policy, Research and International Development, Public Health Wales, Hadyn Ellis Building, Maindy Road, Cardiff, CF24 4HQ UK

**Keywords:** Adverse childhood experiences, Child maltreatment, Mental well-being, Life satisfaction, Prevention

## Abstract

**Background:**

Individuals’ childhood experiences can strongly influence their future health and well-being. Adverse childhood experiences (ACEs) such as abuse and dysfunctional home environments show strong cumulative relationships with physical and mental illness yet less is known about their effects on mental well-being in the general population.

**Methods:**

A nationally representative household survey of English adults (*n* = 3,885) measuring current mental well-being (Short Edinburgh-Warwick Mental Well-being Scale SWEMWBS) and life satisfaction and retrospective exposure to nine ACEs.

**Results:**

Almost half of participants (46.4 %) had suffered at least one ACE and 8.3 % had suffered four or more. Adjusted odds ratios (AORs) for low life satisfaction and low mental well-being increased with the number of ACEs. AORs for low ratings of all individual SWEMWBS components also increased with ACE count, particularly never or rarely feeling close to others. Of individual ACEs, growing up in a household affected by mental illness and suffering sexual abuse had the most relationships with markers of mental well-being.

**Conclusions:**

Childhood adversity has a strong cumulative relationship with adult mental well-being. Comprehensive mental health strategies should incorporate interventions to prevent ACEs and moderate their impacts from the very earliest stages of life.

## Background

Individuals’ childhood experiences are of paramount importance in determining their future outcomes. Research exposing the harmful effects that childhood adversity has on adult physical and mental health has advanced significantly over the past few decades. For instance, the Adverse Childhood Experiences (ACE) framework has provided a mechanism for retrospectively measuring childhood adversities and identifying their impact on health in later life [1]. ACEs include child maltreatment (e.g. physical, sexual and verbal abuse) and broader experiences of household dysfunction, such as witnessing violence in the home, parental separation and growing up in a household affected by substance misuse, mental illness or criminal behaviour. Studies show a dose-responsive relationship between ACEs and poor outcomes, with the more ACEs a person suffers the greater their risks of developing health harming behaviours (e.g. substance misuse, risky sexual behaviour), suffering poor adult health (e.g. obesity, cancer, heart disease) and ultimately premature mortality [1–6].

Much research on the long-term impacts of ACEs has focused on their relationships with mental illness. Thus, studies have found increasing numbers of ACEs to be associated with increasing risks of conditions including depression, anxiety, panic reactions, hallucinations, psychosis and suicide attempt, along with overall psychopathology, psychotropic medication use and treatment for mental disorders [2, 3, 7–11]. However the literature on the impact of ACEs on broader measures of mental health and well-being is less extensive. While definitions vary [12], mental well-being is widely recognised as being more than just the absence of mental illness; incorporating aspects of mental functioning, feelings and behaviours and having been simply described as feeling good and functioning well [13]. Positive mental well-being has been associated with better physical and mental health and with reduced mortality in both healthy and ill populations [14, 15]. Correspondingly, the promotion of mental well-being has become a public and mental health priority both globally and in countries such as the UK [16, 17].

Understanding how different factors impede mental well-being in adults is imperative to investing effectively and efficiently in its promotion. With little longitudinal data available, considerable focus has been placed on the associations between current conditions (e.g. social relationships, residential deprivation, physical exercise, health status) and mental well-being rather than longer-term drivers. However, a US study using the ACE framework found a cumulative relationship between childhood adversity and markers of mental well-being in the general population, including mentally healthy days and life satisfaction [18]. In England, we conducted a pilot ACE study in a local administrative area which found increased odds of low life satisfaction and low mental well-being in adults with increased ACEs [19]. Following this pilot, we undertook a national ACE study of adults across England that included validated measurements of mental well-being and life satisfaction. Here we explore relationships between levels of exposure to adversity during childhood and current mental well-being in adults. Finally, we discuss the convergence between the roots of poor physical health and poor mental well-being in early years and consequently, how poor mental well-being in one generation may adversely impact well-being in the next.

## Methods

A target sample size of 4,000 adult residents of England was established based on the prevalence of ACEs identified in the pilot study [19]. Study inclusion criteria were: aged 18–69 years; resident in a selected LSOA; and cognitively able to participate in a face-to-face interview. Households were selected through random probability sampling stratified by English region (*n* = 10, with inner and outer London treated as two regions) and then by small area deprivation using lower super output areas (LSOAs; geographic areas with a population mean of 1,500) [20]. Within each region, LSOAs were categorised into deciles of deprivation based on their ranking in the 2010 Index of Multiple Deprivation (IMD; a composite measure including 38 indicators relating to economic, social and housing issues) [21]. Two LSOAs were then randomly selected from each decile in each region and for each LSOA between 40 and 120 addresses were randomly selected for inclusion from the Postcode Address File^®^. Sample sizes in each region were proportionate to their population to provide a sample representative of the English population, with a total of 16,000 households initially sampled to account for ineligibility, non-response and non-compliance.

Sampled households were sent a letter prior to researchers visiting providing information on the study and the opportunity to opt out; 771 (4.8 %) households opted out at this stage. Operating under the direction of the research team, a professional survey company visited households on differing days/times (seven days a week, 9:30 am to 8.30 pm) between April and July 2013. The protocol employed by the survey company was to remove households after four attempted visits with no contact. Where contact was made and more than one household member met the inclusion criteria, the eligible resident with the next birthday was selected for interview. Interviewers explained the purpose of the study, outlined its voluntary and anonymous nature and provided a second opportunity for individuals to opt out, with informed consent obtained verbally at the point of interview. Household visits ceased once the target sample size was achieved. Thus, 9,852 of the sampled households were visited of which 7,773 resulted in contact with a resident. Of these households, 2,719 (35.0 %) opted out, 1,044 (13.4 %) were ineligible and 4,010 completed a study questionnaire. Compliance was 59.6 % across eligible occupied households visited and 53.5 % when including those opting out at the letter stage.

The study used an established questionnaire covering demographics, lifestyle behaviours, health status, mental well-being, life satisfaction and exposure to ACEs before the age of 18 [19]. Participants were able to complete the questionnaire through a face-to-face interview using a hand held computer (with sensitive questions self-completed; *n* = 3,852), or to self-complete using paper questionnaires (*n* = 158). Mental well-being was measured using the Short Warwick-Edinburgh Mental Well-being Scale (SWEMWBS) [22], which asks individuals how often over the past two weeks they have been: *feeling optimistic about the future; feeling useful; feeling relaxed; dealing with problems well; thinking clearly; feeling close to other people; able to make up their own mind about things*. Responses are scored from 1 (none of the time) to 5 (all of the time) and an overall mental well-being score is calculated, ranging from 7 (lowest possible mental well-being) to 35 (highest possible mental well-being). Life satisfaction was measured on a scale of 1–10 using the standard question: *All things considered how satisfied are you with your life, with 1 being not at all satisfied and 10 very satisfied* [23]. ACEs were measured using the Centers for Disease Control and Prevention short ACE tool [24] which comprises eleven questions covering nine ACE types: physical abuse; verbal abuse; sexual abuse (three questions); parental separation; exposure to domestic violence; and growing up in a household with mental illness, alcohol abuse, drug abuse or incarceration (for further information see [4]). Ethnicity was recorded using standard UK Census categories [25] and categorised as White, Asian and Other due to small numbers within individual ethnic groups. Respondents were allocated an IMD 2010 quintile of deprivation based on their LSOA of residence. Ethical approval for the study was obtained from Liverpool John Moores University’s Research Ethics Committee and the study adhered to the Declaration of Helsinki.

Analyses were undertaken using SPSS v20. Only individuals with complete data relating to all ACEs, age, sex, ethnicity, and IMD quintile were included in the analysis, resulting in a final sample size of 3,885. Bivariate analyses used chi-squared with backwards conditional logistic regression used to examine independent relationships between ACEs and adult mental well-being and life satisfaction. Consistent with other work including previous ACE studies [1–3] and the World Mental Health Surveys [26–28], the number of ACEs participants reported exposure to was summed into an ACE count (range 0 to 9) and here categorised into four groups for analysis: 0 ACEs (*n* = 2,072), 1 ACE (*n* = 879), 2–3 ACEs (*n* = 594) and 4 + ACEs (*n* = 322). We also explored relationships between outcome variables and individual ACEs, with analysis focusing on those with highly significant relationships. The seven individual components of SWEMWBS were each dichotomised to indicate poor ratings (never or rarely in the last two weeks). Overall SWEMWBS scores and life satisfaction (LS) ratings were dichotomised to indicate low scores as >1 standard deviation (SD) below the mean (SWEMWBS, mean 27.5, SD 4.4, low <23; LS, mean 7.7, SD 1.7, low <6).

## Results

The demographic breakdown of the sample is shown in Table 1. Compared with the English population the sample overrepresented females (55.0 % v 50.3 % in England) and individuals aged 60–69 years (20.7 % v 16.1 %) and underrepresented those aged 18–29 (21.0 % v 24.2 %). There were no differences by deprivation quintile or ethnicity. Just under half of participants reported having suffered at least one ACE (46.4 %) with 15.4 % reporting 2–3 ACEs and 8.3 % 4+ ACEs. The proportion of participants with low measures (never or rarely in the last two weeks) for the individual components of SWEMWBS ranged from 2.5 % (able to make up own mind) to 14.5 % (feeling relaxed). Thirteen percent were categorised as having low SWEMWBS scores (<23) and 11.6 % as having low life satisfaction (score <6; Table 1).

**Table 1:** Sample characteristics and prevalence of low mental well-being and life satisfaction

		% in the last two weeks that have never or rarely been^a^							% with low^b^	
	All	Feeling optimistic	Feeling useful	Feeling relaxed	Dealing well with problems	Thinking clearly	Feeling close to others	Able to make up own mind	SWEMWBS score	LS
n	3,885	3,876	3,882	3,882	3,879	3,883	3,879	3,883	3,868	3,884
All		12.9	8.4	14.5	6.2	3.5	5.3	2.5	13.0	11.6
Age group										
18–29	21.0	9.7	8.7	13.7	6.5	2.5	4.4	3.1	11.2	8.3
30–39	19.9	8.4	7.6	16.7	6.0	3.1	4.3	2.1	11.3	8.3
40–49	20.5	14.2	8.2	17.7	7.0	4.8	6.5	2.8	15.9	15.4
50–59	18.0	19.5	10.0	16.2	6.4	4.7	7.3	4.0	17.1	16.6
60–69	20.7	13.2	7.7	8.7	5.1	2.5	4.0	0.9	10.0	9.8
P		<0.001	0.461	<0.001	0.579	0.015	0.008	0.002	<0.001	<0.001
Gender										
Male	45.0	13.7	9.3	11.6	6.0	3.2	6.1	2.8	12.7	10.8
Female	55.0	12.2	7.7	16.9	6.4	3.7	4.6	2.3	13.3	12.2
P		0.163	0.067	<0.001	0.549	0.397	0.041	0.318	0.604	0.156
Ethnicity										
White	86.3	12.7	8.5	14.6	6.0	3.5	5.2	2.5	12.9	11.4
Asian	7.9	12.4	7.5	14.6	6.8	3.9	5.5	3.2	11.8	10.7
Other	5.7	16.1	9.0	13.5	9.0	3.1	5.4	1.3	16.6	14.4
P		0.321	0.794	0.896	0.178	0.887	0.973	0.384	0.226	0.363
IMD Quintile										
(least deprived)1	20.1	7.4	5.5	13.3	4.1	1.9	3.5	1.7	8.2	7.4
2	19.5	12.1	6.5	15.0	5.7	3.0	3.3	1.6	10.6	8.8
3	19.7	12.6	8.0	13.0	6.6	3.8	4.7	2.5	12.4	8.2
4	19.9	13.6	8.5	14.9	7.0	3.9	6.0	3.4	14.6	14.2
(most deprived) 5	20.7	18.5	13.4	16.4	7.7	4.7	8.7	3.5	19.1	18.8
P		<0.001	<0.001	0.291	0.033	0.034	<0.001	0.036	<0.001	<0.001
ACE count										
0	53.6	10.7	6.5	10.9	4.8	2.5	3.3	2.0	9.5	7.9
1	22.7	12.9	6.9	14.1	5.7	3.5	4.9	2.4	12.1	11.7
2–3	15.4	15.3	11.7	20.1	8.2	4.2	8.6	3.5	17.2	16.1
4+	8.3	22.3	18.9	28.8	13.3	8.0	13.0	4.6	30.7	26.6
P		<0.001	<0.001	<0.001	<0.001	<0.001	<0.001	0.012	<0.001	<0.001

^a^Variables represent the individual component questions in the SWEMWBS scale. ^b^SWEMWBS (Short Warwick-Edinburgh Mental Well-being Scale) score <23; LS (life satisfaction) rating <6

Low SWEMWBS scores and LS were both associated with age, being most prevalent in the 50–59 year age group (Table 1). Significant relationships with age were also seen for all individual SWEMWBS components except feeling useful and dealing with problems. There were no relationships between gender and LS or overall SWEMWBS score, although among the individual SWEMWBS components more females had low scores for feeling relaxed and more males for feeling close to others. There were no significant relationships between ethnicity and either low SWEMWBS score or low LS. However both outcomes increased with deprivation, as did low levels of all individual SWEMWBS components except feeling relaxed.

There were strong associations between ACE count and all markers of low mental well-being. Thus the prevalence of low SWEMWBS score tripled from 9.5 % in those with 0 ACEs to 30.7 % in those with 4+ ACEs, while the prevalence of low LS more than tripled from 7.9 to 26.6 % respectively. These significant relationships remained after controlling for confounders in logistic regression analysis with adjusted odds ratios (AORs) for low SWEMWBS score and low LS increasing with ACE count and reaching 3.9 for both outcomes in those with 4+ ACEs (compared with 0 ACEs; Table 2). Importantly, while associations between both outcomes and age also remained in LR, running separate models for each age group showed the relationships between high ACE count and low mental well-being to be consistent across age groups. Thus, compared with individuals with no ACEs, AORs for low SWEBWBS scores in those with 4+ ACEs ranged from 3.08 in both 18–29 year olds (95 % CIs, 1.56–6.07) and 30–39 year olds (95 % CIs 1.66–5.72) to 5.34 (95 % CIs 2.10–13.57) in 60–69 year olds (all *p* < 0.001) and for low LS from 2.54 (95 % CIs 1.09–5.90, *p* = 0.030) in 18–29 year olds to 11.20 (95 % CIs 4.43–28.29, *p* < 0.001) in 60–69 year olds.

**Table 2:** Adjusted odds ratios for low mental well-being and life satisfaction

		Low^a^					
		SWEMWBS score			Life satisfaction		
		AOR	95 % CIs	*P*	AOR	95 % CIs	*P*
Age	18–29 (Ref)			***			***
	30–39	1.049	0.763–1.444	ns	1.057	0.734–1.521	ns
	40–49	1.609	1.196–2.165	**	2.227	1.612–3.076	***
	50–59	1.893	1.398–2.562	***	2.683	1.992–3.774	***
	60–69	1.100	0.795–1.523	ns	1.572	1.108–2.229	*
Deprivation quintile	1 (least deprived; Ref)			***			***
	2	1.244	0.877–1.765	ns	1.135	0.783–1.647	ns
	3	1.517	1.081–2.130	*	1.073	0.736–1.564	ns
	4	1.770	1.270–2.465	**	1.992	1.414–2.808	***
	5 (most deprived)	2.382	1.734–3.273	***	2.714	1.952–3.774	***
ACE count	0 (Ref)			***			***
	1	1.350	1.048–1.739	*	1.636	1.256–2.132	***
	2–3	1.946	1.497–2.529	***	2.235	1.696–2.947	***
	4+	3.856	2.896–5.134	***	3.893	2.867–5.286	***

^a^SWEMWBS (Short Warwick-Edinburgh Mental Well-being Scale) score <23; Life satisfaction rating <6. *AOR* adjusted odds ratio; *95 % CIs* 95 % confidence intervals; *Ref* reference category; *P < 0.05, **P < 0.01, ***P < 0.001, *ns* not significant. Analyses used backward conditional logistic regression. Gender and ethnicity were also entered into the model but were not significantly related to low SWEMWBS score or low life satisfaction (data not shown)

**Fig. 1:**
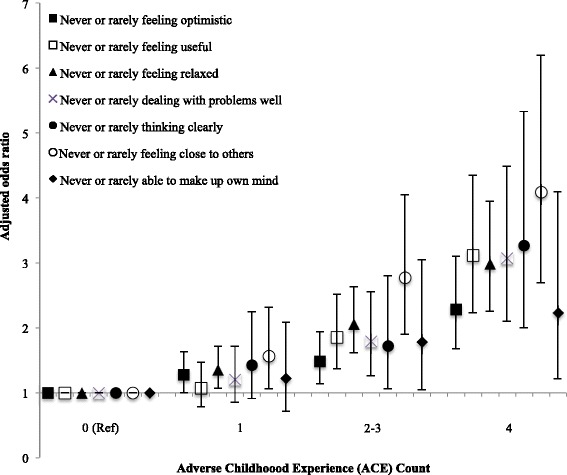
Relationship between adverse childhood experience count and components of poor adult mental well-being (adjusted odds ratios and 95 % confidence intervals). Variables represent the individual component questions in the SWEMWBS scale. Adjusted odds ratios were calculated using logistic regression analysis. Additional independent variables included in the logistic regression were age, gender, deprivation and ethnicity. All relationships are significant with poor mental well-being components positively related to increasing ACE count (*p* < 0.001, except ‘ability to make up own mind where *p* < 0.05). Ref = reference category

Figure 1 presents AORs for low scores for each component of SWEMWBS by increasing ACE count (all ages). All relationships were significant and cumulative with AORs for those with 4+ ACEs (compared with 0 ACEs) ranging from 2.23 (95 % CIs 1.22–4.10) for never or rarely being able to make up one’s own mind to 4.09 (2.70–6.20) for never or rarely feeling close to others.

Table 3 shows the relationships between measures of mental well-being and the nine individual ACEs examined. Physical, sexual and emotional abuse, witnessing domestic violence, and living in a household affected by mental illness or drug abuse were significantly associated with low levels of all mental well-being measures and household alcohol misuse and incarceration with low levels of all except the ability to make one’s own mind up about things. However parental separation or divorce was only associated with two of the seven SWEMWBS components (feeling useful, feeling relaxed) and an overall low SWEMWBS score. For each marker of mental well-being, a logistic regression model was run that included individual ACE types significantly related to the marker (in bivariate analysis, see Table 3) and demographic variables. Here, household mental illness was found to have independent relationships with the most mental well-being marker, being associated with all except the SWEMWBS component of feeling relaxed (Table 4). Childhood sexual abuse was associated with all except the SWEMWBS components of feeling useful and feeling close to others. Emotional and physical abuse each had independent relationships with five of the nine measures and household alcohol problems with four. Feeling close to others (the SWEMWBS component with the strongest relationship with ACE count; Fig. 1), was independently associated with household mental illness, emotional abuse and physical abuse.

**Table 3:** Bivariate relationships between mental well-being measures and individual adverse childhood experience types

ACE	All	In the last two weeks, % never or rarely^a^:							% with low^b^:	
		Feeling optimistic	Feeling useful	Feeling relaxed	Dealing well with problems	Thinking clearly	Feeling close to others	Able to make up own mind	SWEMWBS score	LS
Physical abuse										
No	85.7	11.6	7.2	13.0	5.6	3.0	4.2	2.3	11.1	9.6
Yes	14.3	20.5	15.6	23.9	10.1	6.5	11.9	3.9	24.2	23.2
P		<0.001	<0.001	<0.001	<0.001	<0.001	<0.001	0.020	<0.001	<0.001
Emotional abuse										
No	82.7	11.9	7.1	12.6	5.5	3.0	3.9	2.2	10.7	9.3
Yes	17.3	17.6	14.7	24.0	9.8	5.8	11.9	4.0	24.0	22.2
P		<0.001	<0.001	<0.001	<0.001	<0.001	<0.001	0.007	<0.001	<0.001
Sexual abuse										
No	93.8	12.2	7.9	13.6	5.7	3.0	4.8	2.3	11.7	10.4
Yes	6.2	23.4	16.7	28.9	14.2	10.9	13.0	6.7	33.2	28.9
P		<0.001	<0.001	<0.001	<0.001	<0.001	<0.001	<0.001	<0.001	<0.001
Parental separation										
No	77.4	12.9	7.8	13.2	6.1	3.5	5.2	2.5	12.4	11.1
Yes	22.6	12.8	10.5	19.2	6.7	3.3	5.4	2.7	15.1	13.2
P		0.906	0.013	<0.001	0.486	0.744	0.880	0.657	0.040	0.084
Domestic violence										
No	87.9	12.2	8.0	13.3	5.6	3.2	4.7	2.3	11.8	10.7
Yes	12.1	18.1	11.7	23.2	10.4	5.7	9.2	4.0	21.6	17.9
P		<0.001	0.006	<0.001	<0.001	0.004	<0.001	0.025	<0.001	<0.001
Mental illness										
No	87.9	12.0	7.6	13.3	5.4	3.0	4.3	2.2	11.2	10.2
Yes	12.1	19.1	14.7	23.4	12.4	7.2	12.0	4.9	25.9	21.5
P		<0.001	<0.001	<0.001	<0.001	<0.001	<0.001	<0.001	<0.001	<0.001
Alcohol misuse										
No	90.9	12.3	7.7	13.4	5.5	3.2	4.8	2.4	11.9	10.3
Yes	9.1	19.0	16.1	25.8	13.0	6.5	9.9	4.0	24.4	23.8
P		<0.001	<0.001	<0.001	<0.001	0.001	<0.001	0.070	<0.001	<0.001
Drug misuse										
No	96.1	12.5	8.2	14.2	5.8	3.2	5.0	2.4	12.6	11.2
Yes	3.9	20.9	15.0	22.9	16.3	11.1	11.2	5.2	23.7	19.6
P		0.002	0.003	0.003	<0.001	<0.001	0.001	0.030	<0.001	0.001
Incarceration										
No	95.9	12.5	8.1	13.9	5.9	3.3	5.0	2.5	12.5	11.1
Yes	4.1	21.5	17.1	28.5	13.9	7.0	12.0	2.5	24.7	22.2
P		0.001	<0.001	<0.001	<0.001	0.015	<0.001	0.995	<0.001	<0.001

^a^Variables represent the individual component questions in the SWEMWBS scale. ^b^SWEMWBS (Short Warwick-Edinburgh Mental Well-being Scale) score <23; LS (life satisfaction) rating <6

**Table 4:** Adjusted odds ratios for low ratings on SWEMWBS components, low overall SWEMWBS scores and low LS in those reporting individual adverse childhood experience types

ACE		In the last two weeks, never or rarely^a^							Low^b^	
		Feeling optimistic	Feeling useful	Feeling relaxed	Dealing well with problems	Thinking clearly	Feeling close to others	Able to make up own mind	SWEMWBS score	LS
Physical abuse	AOR	1.50	1.53	1.39			1.55			1.49
	95 % CIs	1.16–1.94	1.11–2.13	1.06–1.83			1.05–2.28			1.11–2.00
	P	**	*	*	ns	ns	*	ns	ns	**
Emotional abuse	AOR		1.46	1.47			2.12		1.73	1.54
	95 % CIs		1.06–2.00	1.13–1.89			1.46–3.08		1.36–2.20	1.16–2.05
	P	ns	*	**	ns	ns	***	ns	***	**
Sexual abuse	AOR	1.55		1.52	1.82	2.68		2.5	2.30	1.79
	95 % CIs	1.10–2.20		1.08–2.12	1.19–2.79	1.64–4.38		1.40–4.49	1.67–3.18	1.27–2.54
	P	*	ns	*	**	***	ns	**	***	**
Parental separation	AOR	na			na	na	na	na		na
	95 % CIs									
	P	-	ns	ns					ns	
Domestic violence	AOR									
	95 % CIs									
	P	ns	ns	ns	ns	ns	ns	ns	ns	ns
Mental illness	AOR	1.43	1.44		1.78	1.79	2.04	1.85	1.97	1.47
	95 % CIs	1.09–1.88	1.04–2.00		1.25–2.53	1.15–2.78	1.42–2.94	1.12–3.07	1.52–2.55	1.09–1.97
	P	*	*	ns	**	*	***	*	***	*
Alcohol problem	AOR		1.46	1.41	1.56			na		1.52
	95 % CIs		1.02–2.07	1.06–1.89	1.04–2.34					1.11–2.08
	P	ns	*	*	*	ns	ns		ns	*
Drug misuse	AOR				1.76	2.45				
	95 % CIs				1.06–2.93	1.34–4.46				
	P	ns	ns	ns	*	**	ns	ns	ns	ns
Incarceration	AOR			1.53				na		
	95 % CIs			1.03–2.26						
	P	ns	ns	*	ns	ns	ns		ns	ns

^a^Variables represent the individual component questions in the SWEMWBS scale. ^b^SWEMWBS (Short Warwick-Edinburgh Mental Well-being Scale) score <23; LS (life satisfaction) rating <6. Analysis used backward conditional logistic regression. Separate models were run for each SWEMWBS component, low SWEMWBS score and low LS. Models included ACE types significantly related to each outcome in bivariate analysis along with age, gender, IMD quintile of deprivation and ethnicity. *na* not applicable; variable not included in the model due to lack of relationship in bivariate analyses. For each ACE, the reference group is those that did not report the ACE. *P < 0.05, **P < 0.01, ***P < 0.001, *ns* not significant

## Discussion

Promoting mental well-being has become a major public health priority as recognition of the links between well-being and broader health and social outcomes has grown. This has contributed to the emergence of broader policy approaches to mental health, both globally and nationally, that incorporate population-level prevention and promotion activity alongside traditional therapeutic responses to mental illness [16, 17]. In England, motivation for increased investment in mental well-being promotion has centred around the notion that interventions to improve mental well-being at a population level could produce greater benefits than those to prevent mental illness in at-risk populations [29, 30]. However, the evidence base on which such approaches are based is being questioned as broader measurements and studies of mental well-being emerge [12]. Thus, existing studies have largely associated mental well-being in adults with factors linked to their current circumstances, such as employment, residential deprivation, social participation, physical exercise, relationship satisfaction and health status [31]. Correspondingly, interventions have often focused on promoting individual behavioural change through, for example, increasing social connectedness and physical activity [32, 33]. A life course perspective that incorporates the longer-term impact of childhood adversity has largely been absent from discussions on mental well-being.

Using a randomly selected national household sample of English adults, our study found a strong cumulative relationship between childhood adversities and two widely used measures of mental well-being. The more ACEs participants reported having suffered during their childhood the more likely they were to report low SWEMWBS scores and low life satisfaction (Table 1). These relationships remained after controlling for demographics, with odds of poor outcomes for both measures being elevated in those with even a single ACE and almost four times higher in those with four or more ACEs (compared with those with no ACEs; Table 2). We also found ACE count to be independently related to each of the seven individual components of SWEMWBS; individuals with higher ACE counts were more likely to report never or rarely (in the last two weeks) feeling optimistic, useful, relaxed or close to others, dealing with problems well, thinking clearly and being able to make up one’s own mind (Fig. 1).

A variety of mechanisms link ACEs to poor adult mental well-being. Critically, maltreatment and other stressors in childhood can affect brain development and have harmful, lasting effects on emotional functioning [2, 34]. Children who are maltreated can develop attachment difficulties, including poor emotional regulation, lack of trust and fear of getting close to other people. They can also form negative self-images, lack self-worth and suffer feelings of incompetence, all of which can be retained into adulthood [2, 34, 35]. The relationships between ACEs and factors including poor educational attainment and the development of health-damaging behaviours mean that individuals who suffer ACEs can also face a range of risk factors for poor mental well-being in adulthood, such as poor health, low employment and social deprivation [2, 4, 36]. These effects can contribute to cycles of adversity and poor mental well-being whereby individuals that grew up in adverse conditions are less able to provide optimum childhood environments for their own offspring [37]. Here, and consistent with previous work [38], the SWEMWBS component with the strongest relationship with ACE count was never or rarely feeling close to others. Children whose parents show poor relationships with them are at greater risks of ACEs [39], thus individuals who cannot feel close to others as a result of their own ACE history may subsequently be more likely to expose their own children to ACEs. These relationships may also have implications for the implementation and effectiveness of interventions to improve mental well-being through social connectedness.

While analysis based on ACE count highlights the cumulative impact of childhood adversity on mental well-being, it is also useful to explore which ACEs may have particular effects. All ACE types showed significant bivariate relationships with low SWEMWEBS scores, and all except parental separation/divorce with low life satisfaction and most individual SWEMWBS components. In multivariate analyses, however, the ACEs with the most independent relationships with markers of low mental well-being were growing up in a household with someone affected by mental illness and suffering childhood sexual abuse.

The links between growing up in a household affected by mental illness in childhood and low mental well-being in adulthood may in part reflect genetic risk factors that make the offspring of individuals with mental disorders susceptible to poor mental health themselves [40]; although genetic explanations for the transmission of mental disorders are disputed [41]. Thus, parental mental illness can have broader impacts on children’s social and emotional development when parenting practices are affected by factors such as low emotional warmth, reduced responsiveness, impaired attention and unpredictable behavioural patterns [42]. An extensive body of research provides evidence that exposure to childhood adversity such as parental stress, disrupted care patterns and abuse increases risks of mental illness [43], while studies are increasingly identifying how exposure to such adversity can trigger epigenetic modifications to gene expressions, altering brain structure, stress reactivity and consequently vulnerability to both mental and physical ill health [44]. Childhood sexual abuse can have particularly damaging effects on individuals’ emotional development, having been linked to feelings of shame and self-blame, powerlessness, inappropriate sexual beliefs and difficulties forming and maintaining intimate relationships [45, 46]. Correspondingly research has identified strong relationships between childhood sexual abuse and adult mental illness [11]. For example, in England sexual abuse in childhood has been attributed to 11 % of all common mental disorders, along with 7 % of alcohol dependence disorders, 10 % of drug dependence disorders, 15 % of eating disorders and 17 % of post-traumatic stress disorders [47].

The WHO Mental Health Action Plan 2013–2020 incorporates the promotion of mental well-being as part of its overarching goal: highlighting the need for a life course approach that intervenes early to prevent mental health difficulties; recognising the importance of reducing violence; and emphasising the importance of services being responsive to the needs of survivors of violence [17]. Interventions that seek to reduce ACEs, develop parenting skills and promote resilience in children should thus be considered essential elements in comprehensive mental health strategies. Starting at the very earliest stages of life, these can include measures to train midwives, health visitors and other early years professionals to enquire about parental mental well-being and identify and treat post-natal depression and other mental health concerns [48]. The ante- and post-natal periods also offer the opportunity to identify and address a broader range of ACEs including parental substance use and domestic violence as well as to increase parenting skills and knowledge. Effective interventions include home visiting and parenting programmes that promote parent-child bonding and develop parenting skills, along with social and emotional development programmes that strengthen life skills and thus resilience in children [49, 50]. Measures should also be taken to ensure service providers across a broad range of disciplines are cognisant of the lasting damage that ACEs place on mental well-being and wider health and social outcomes, and are trained to recognise and respond appropriately to clients with adverse backgrounds [51]. In particular, professionals in mental health services should be trained to routinely enquire about childhood experiences during client assessments. Studies suggest such enquiry is often lacking, with mental health treatment typically based on a medical model that focuses on biological factors and ignores the profound influence of socio-environmental experiences on brain development and functioning [52, 53].

While the ACE methodology has been widely employed [54] it remains vulnerable to issues associated with any cross-sectional and retrospective survey with, for example, results relying on accurate recall and willingness to report ACEs. While adults with low mental well-being may have more negative perceptions of their childhoods, studies suggest false-positive reports of ACEs are rare [55]. Measures of current mental well-being and life satisfaction were also self-reported and therefore vulnerable to subjectivity, while the exclusion of individuals cognitively unable to participate in a face-to-face survey may have created bias in our sample. The dichotomisation of well-being scales may also have resulted in loss of information, although we used a consistent method to identify low mental well-being of greater than one SD from the sample mean. We used a recognised tool to measure nine important ACEs yet other common adversities such as neglect, bullying and parental death were not recorded. We explored the independent associations between outcome variables and both ACE counts and individual ACEs. However, we had insufficient sample size to look at how interactions between the individual ACE types, different combinations of ACEs and demographics may have resulted in different relationships with mental wellbeing. Such limitations aside our analyses did include multiple statistical analyses potentially increasing risks of type I errors. Consequently, while we have presented all figures for transparency, discussion has focused on highly significant results [56]. Finally, our study did not measure resilience resources [57], and developing understanding of factors that promote resiliency in those affected by ACEs would be an important future research priority.

## Conclusions

While the high prevalence of mental disorders in the most vulnerable children (e.g. those in child protection systems) and the continued risks of mental illness in adults who suffered ACEs are widely recognised, data linking childhood adversity to the development and persistence of low mental well-being in the broader population is scarce. Our study suggests that almost half of the general English population have experienced at least one ACE and over one in twelve have suffered four or more ACEs. Such childhood adversity places individuals at significantly increased risk of low mental well-being and may have implications for the implementation and success of interventions that seek to promote mental well-being in the general population. The strong links between ACEs and adult mental well-being emphasise the need for a life course approach to mental health with the drivers of poor mental and physical health outcomes rooted together in childhood issues. Many of the ACEs that impact on children’s long term health and well-being are linked to familial behaviours and mental health (e.g. mental illness, substance abuse, violent and aggressive behaviour) suggesting that the mental health impacts of ACEs are what pushes much of their cyclical nature. A life course approach suggests that preventing ACEs would contribute to better physical and mental health from childhood through to old age and thus improve mental well-being in future generations.

## Availability of data and materials

Data sets and other materials used in this article can be accessed by request to Professor Karen Hughes.

## Abbreviations

ACE: adverse childhood experience
AOR: adjusted odds ratio
CI: confidence interval
IMD: Index of Multiple Deprivation
LS: life satisfaction
LSOA: lower super output area
SWEMWBS: Short Warwick and Edinburgh Mental Well-being Scale
